# Adiponectin Enhances Intercellular Adhesion Molecule-1 Expression and Promotes Monocyte Adhesion in Human Synovial Fibroblasts

**DOI:** 10.1371/journal.pone.0092741

**Published:** 2014-03-25

**Authors:** Hsien-Te Chen, Hsi-Kai Tsou, Jui-Chieh Chen, James Meng-Kun Shih, Yen-Jen Chen, Chih-Hsin Tang

**Affiliations:** 1 School of Chinese Medicine, College of Chinese Medicine, China Medical University, Taichung, Taiwan; 2 Department of Orthopaedic Surgery, China Medical University Hospital, Taichung, Taiwan; 3 Department of Neurosurgery, Taichung Veterans General Hospital, Taichung, Taiwan; 4 Department of Early Childhood Care and Education, Jen-Teh Junior College of Medicine, Nursing and Management, Miaoli County, Taiwan; 5 Department of Orthopaedic Surgery, Lin Sen Hospital, Taichung, Taiwan; 6 School of Medicine, China Medical University, Taichung, Taiwan; 7 Graduate Institute of Basic Medical Science, China Medical University, Taichung, Taiwan; 8 Department of Biotechnology, College of Health Science, Asia University, Taichung, Taiwan; 9 National Institute of Cancer Research, National Health Research Institutes, Miaoli County, Zhunan, Taiwan; Chang Gung University, Taiwan

## Abstract

Adiponectin is a protein hormone secreted predominantly by differentiated adipocytes and is involved in energy homeostasis. Adiponectin expression is significantly high in the synovial fluid of patients with osteoarthritis (OA). Intercellular adhesion molecule-1 (ICAM-1) is an important adhesion molecule that mediates monocyte adhesion and infiltration during OA pathogenesis. Adiponectin-induced expression of ICAM-1 in human OA synovial fibroblasts (OASFs) was examined by using qPCR, flow cytometry and western blotting. The intracellular signaling pathways were investigated by pretreated with inhibitors or transfection with siRNA. The monocyte THP-1 cell line was used for an adhesion assay with OASFs. Stimulation of OASFs with adiponectin induced ICAM-1 expression. Pretreatment with AMP-activated protein kinase (AMPK) inhibitors (AraA and compound C) or transfection with siRNA against AMPKα1 and two AMPK upstream activator- liver kinase B1 (LKB1) and calmodulin-dependent protein kinase II (CaMKII) diminished the adiponectin-induced ICAM-1 expression. Stimulation of OASFs with adiponectin increased phosphorylation of LKB1, CaMKII, AMPK, and c-Jun, resulting in c-Jun binding to AP-1 element of ICAM-1 promoter. In addition, adiponectin-induced activation of the LKB1/CaMKII, AMPK, and AP-1 pathway increased the adhesion of monocytes to the OASF monolayer. Our results suggest that adiponectin increases ICAM-1 expression in human OASFs via the LKB1/CaMKII, AMPK, c-Jun, and AP-1 signaling pathway. Adiponectin-induced ICAM-1 expression promoted the adhesion of monocytes to human OASFs. These findings may provide a better understanding of the pathogenesis of OA and can utilize this knowledge to design a new therapeutic strategy.

## Introduction

Osteoarthritis (OA) is the most common chronic degenerative joint disorder in elderly individuals, which is often characterized by infiltration of inflammatory cells and production of multiple potent inflammatory mediators and matrix-degrading proteinases in synovium, leading to disabling pain, stiffness, cartilage breakdown, and a loss of joint function [1]. To date, the etiology of OA is still not fully understood. Nevertheless, emerging evidence has revealed that adipose tissue is capable of secreting a number of adipokines, which have a critical role in the development and progression of OA [2]–[6].

Adiponectin (also known as Acrp30, AdipoQ, and GBP28), one of the most abundant adipokines, is highly expressed in the synovial fluid of patients with OA and closely associated with the severity [7]–[10]. Previous studies showed that adiponectin could be expressed not only by articular adipocytes but also by synovial fibroblasts [11]. In addition, adiponectin receptors have also been identified on the surface of synovial fibroblasts, which are necessary to exert the adiponectin-dependent signals to increase the production of cartilage-degrading matrix metalloproteinase (MMP) enzymes, cytokines and prostaglandin E2 [11]–[13]. Besides release of inflammatory mediators, infiltration of inflammatory cells has also been detected in the inflamed synovium of OA patients, which plays a critical role in persistent inflammation and joint destruction [14]. The movement of mononuclear cells into the inflammatory sites is regulated by adhesion molecules, such as intercellular adhesion molecule-1 (ICAM-1).

ICAM-1 is an inducible surface glycoprotein that belongs to the immunoglobulin superfamily and mediates adhesion-dependent cell-to-cell interactions [15], [16]. The extracellular domain of ICAM-1 plays a crucial role in migration of leukocytes out of blood vessels into sites of inflammation [17]. More recently, a study further demonstrated that tumor-associated fibroblasts isolated from tumor tissues exhibit increased ICAM-1 expression and affinity for monocytes [18]. Up-regulation of ICAM-1 has been shown in synovium of OA patients, which may be an important regulator of leukocyte recruitment into the synovial tissue [19], [20]. Furthermore, reducing the levels of ICAM-1 in synovial fluid also proposed effective method to suppress the inflammatory response and to ameliorate symptoms of physiological distress in OA [21], [22].

Although the roles of adiponectin have emerged as key a regulator of immune responses and inflammatory arthritis, little is known about the mechanisms underlying the interaction between monocytes and human OASFs by which adiponectin induce ICAM-1 expression. In the present study, we explored the possible intracellular signaling pathways involved in adiponectin-induced ICAM-1 expression in human OASFs.

## Materials and Methods

### Material

Rabbit polyclonal antibodies specific for ICAM-1, p-AMPK, AMPK, p-LKB1, LKB1, p-CaMKII, CaMKII, p-c-Jun, c-Jun, and β-actin, anti-mouse and anti-rabbit IgG-conjugated horseradish peroxidase, and Protein A/G beads were purchased from Santa Cruz Biotechnology (Santa Cruz, CA, USA). Compound C and adenosine-9-β-d-arabino-furanoside (AraA) were purchased from Calbiochem (San Diego, CA). Human full-length adiponectin was purchased from R&D Systems (Minneapolis, MN). The AP-1 luciferase plasmid was purchased from Stratagene (La Jolla, CA). The pSV-β-galactosidase vector and luciferase assay kit were purchased from Promega (Madison, MA). All other chemicals were purchased from Sigma-Aldrich (St. Louis, MO).

### Cell cultures

Human synovial fibroblasts were isolated using collagenase treatment of synovial tissues obtained from knee replacement surgeries of 18 patients with OA. OASFs were isolated, cultured, and characterized as previously described [23], [24]. Experiments were performed using cells grown in vitro for 3–6 passages. The study protocol was approved by the Institutional Review Board of China Medical University Hospital, and all subjects gave informed written consent before enrollment. THP-1, a human leukemia cell line of the monocyte/macrophage lineage, was obtained from the American Type Culture Collection (Manassas, VA, USA) and grown in RPMI-1640 medium with 10% fetal bovine serum.

### Quantitative real-time PCR

Total RNA was extracted from OASFs using a TRIzol kit (MDBio Inc., Taipei, Taiwan). The reverse transcription reaction was performed from 2 μg of total RNA using M-MLV reverse transcriptase (Invitrogen) according to the manufacturer's instructions. The quantitative real-time PCR (qPCR) analysis was carried out using Taqman one-step PCR Master Mix (Applied Biosystems, Foster City, CA). The cDNA templates (2 μl) were added per 25-μl reaction with sequence-specific primers and Taqman probes. All target gene primers and probes were purchased commercially (β-actin was used as internal control) (Applied Biosystems). The qPCR assays were carried out in triplicate using a StepOnePlus sequence detection system. Amplification curves were generated with an initial denaturing step at 95°C for10 min, followed by 40 cycles at 95°C for 15 s and 60°C for 60 s. The threshold was set above the non-template control background and within the linear phase of the target gene amplification to calculate the cycle number at which the transcript was detected (denoted CT). Reactions were normalized to copies of β-actin mRNA within the same sample using the −ΔΔCT method. The levels of mRNA are expressed as the fold change in expression compared with that of controls.

### Western blot analysis

Cells were lysed in RIPA buffer containing protease inhibitor cocktail. Protein concentration was determined by the BCA assay (Pierce). Proteins (30 μg) were resolved on SDS-PAGE and transferred to immobilon polyvinyldifluoride (PVDF) membranes. The blots were blocked with 5% BSA for 1 h at room temperature and then probed with rabbit anti-human antibodies against ICAM-1, p-AMPK, AMPK, p-CaMKII, CaMKII, p-LKB1, or LKB1 (1∶1000) for 1 h at room temperature. β-actin was used as an internal control of protein loading. After three washes, the blots were subsequently incubated with the appropriate secondary antibodies conjugated to horseradish peroxidase. Membranes were then washed and bound antibodies were visualized using ECL reagents (PerkinElmer, MA, USA) and autoradiography.

### Flow cytometry analysis

Human synovial fibroblasts were seeded in six-well plates. The cells were then washed with PBS and detached with trypsin at 37°C. After fixation with 1% paraformaldehyde for 10 min at room temperature, cells were resuspended in PBS with mouse anti-human antibody against ICAM-1 (1∶100) for 1 h at 4°C. Cells were then washed again and incubated with FITC-conjugated goat anti-mouse secondary IgG (1∶100; Leinco Technologies Inc., St. Louis, MO, USA) for 45 min and analyzed by flow cytometry using FACS Calibur (10,000 cells were collected for each experiment) and CellQuest software (BD Biosciences).

### Transfection of siRNAs

ON-TARGETplus siRNA of AMPKα1, CaMKII, LKB1, c-Jun, c-fos, and control were purchased from Dharmacon Research (Lafayette, CO). Transient transfection of siRNAs (100 nM) was carried out using DharmaFECT1 transfection reagent, according to the manufacturer's instructions.

### Chromatin immunoprecipitation assay

Chromatin immunoprecipitation (ChIP) analysis was performed as described previously [25]. Briefly, the DNA/protein complex was immunoprecipitated by protein G-agarose beads with anti-c-Jun monoclonal antibody (mAb). After incubation, the beads were washed with the low-salt wash buffer, the high-salt wash buffer, the LiCl wash buffer and finally two times with Tris-EDTA buffer. The bound protein was eluted with elution buffer containing 1% SDS and 100 mM NaHCO3. The crosslinks were reversed by overnight incubation at 65°C. The DNA was then extracted with phenol-chloroform. The purified DNA pellet was subjected to PCR. PCR products were then resolved by 1.5% agarose gel electrophoresis and visualized by UV transillumination. The primers 5′-AGACCTTAGCGCGGTGTAGA-3′ and 5′-AGTAGCAGAGGAGCTCAGCG-3′ were utilized to amplify across the ICAM-1 promoter region (−346 to −24).

### Reporter assay

Human OASF cells were transfected with a reporter plasmid (ICAM-1 luciferase plasmid or AP-1 luciferase) using Lipofectamine 2000 (Invitrogen) according to the manufacturer's recommendations. At 24 h after transfection, the cells were exposed to various doses (0.3–3 μM/ml) of adiponectin for 24 h or pretreated with inhibitors for 30 min, and then, adiponectin or vehicle was added for 24 h. Cell extracts were then prepared, and luciferase and β-galactosidase activities were measured.

### Cell adhesion assay

THP-1 cells were labeled with BCECF-AM (10 μM) at 37°C for 1 h in RPMI-1640 medium and subsequently washed by centrifugation. OASFs grown on glass coverslips were incubated with adiponectin for 6 h. Confluent adiponectin-treated OASFs were incubated with THP-1 cells (2×10^6^ cells/ml) at 37°C for 1 h. Non-adherent THP-1 cells were then removed and gently washed with PBS. The number of adherent THP-1 cells was counted in four randomly chosen fields per well at a high magnification of 200× using a fluorescence microscope (Zeiss, Axiovert 200 M).

### Statistics

The values reported are means ± S.E. Statistical comparisons between two samples were performed using the Student's *t*-test. Statistical comparisons of more than two groups were performed using one-way analysis of variance (ANOVA) with a Bonferroni's *post-hoc* test. In all cases, a p-value of <0.05 was considered significant.

## Results

### Adiponectin induces ICAM-1 expression in human synovial fibroblasts

**Figure 1:**
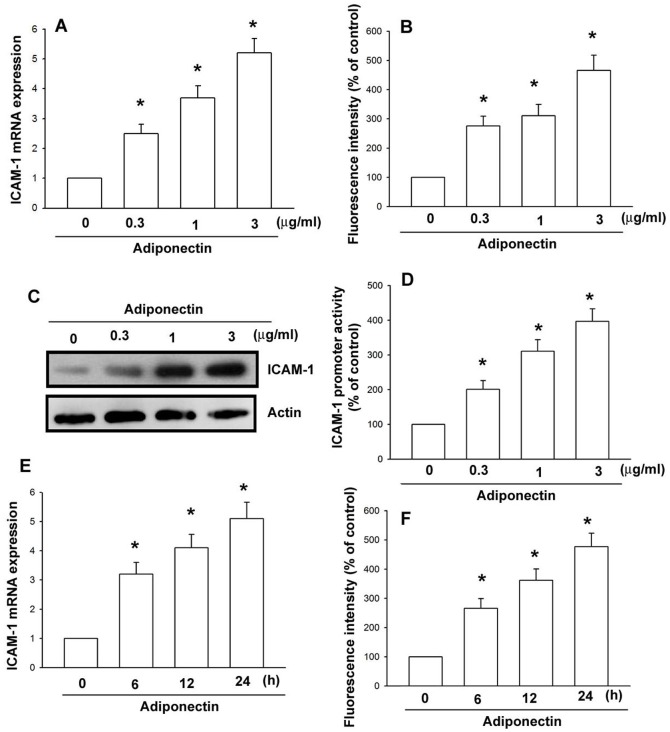
Adiponectin increases ICAM-1 expression. OASFs were incubated with various concentrations of adiponectin for 24-1 mRNA (A), cell surface (B), and protein expression (C) were examined by qPCR, flow cytometry, and western blotting. (D) OASFs were transfected with ICAM-1 promoter-luciferase construct to examine the adiponectin-induced promoter activity in a dose-dependent manner. (E, F) OASFs were incubated with adiponectin (3 μg/ml) for the indicated time intervals. The levels of ICAM-1 mRNA (E) and cell surface (F) expression were examined by qPCR and flow cytometry. Results are expressed as the mean ± S.E. *p<0.05, compared to basal expression levels. #*p*<0.05, compared to expression levels in the adiponectin-treated group.

We initially assessed the effects of adiponectin on the expression of ICAM-1 in human OASFs. The treatment of OASFs with adiponectin resulted in a dose-dependent increase in mRNA and cell surface ICAM-1 expression, as assessed by qPCR (Fig. 1A) and flow cytometry (Fig. 1B). The expression of ICAM-1 was further validated by western blot analysis (Fig. 1C). To clarify whether adiponectin is able to stimulate activation of ICAM-1 promoter in human synovial fibroblasts cells, the human ICAM-1 promoter-luciferase construct was transfected into cells to examine the adiponectin-induced promoter activity. As shown in Fig. 1D, adiponectin increased ICAM-1 promoter activity in a dose-dependent manner. Furthermore, the expression levels of ICAM-1 were also increased in OASFs after treatment with adiponectin in a time dependent (Fig. 1E, F). These data indicate that adiponectin increases ICAM-1 expression in human OASFs.

### AP-1 is involved in the adiponectin-mediated increase of ICAM-1 expression

**Figure 2:**
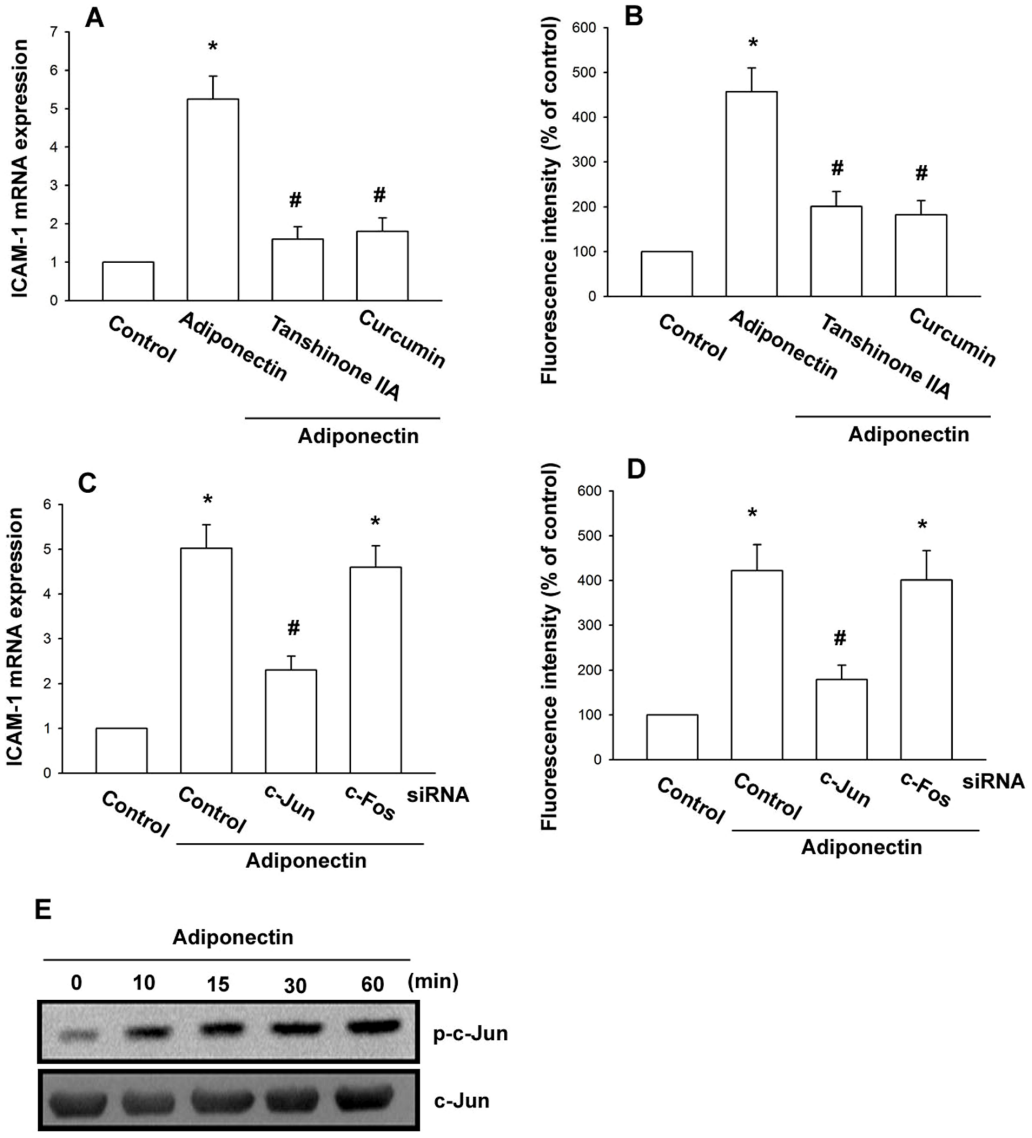
AP-1 is involved in the potentiation of ICAM-1 expression by adiponectin. OASFs were pretreated for 30(3 μM) or tanshinone IIA (5 μM) followed by stimulation with adiponectin (3 μg/ml) for 24 h, and ICAM-1 expression was examined by qPCR (A) and flow cytometry (B). OASFs were transfected with c-Jun siRNA or c-fos siRNA for 24 h followed by stimulation with adiponectin (3 μg/ml) for 24 h, and ICAM-1 expression was examined by qPCR (C) and flow cytometry (D). (E) OASFs were incubated with adiponectin (3 μg/ml) for the indicated time intervals and c-Jun phosphorylation was determined by western blot. Results are expressed as the mean ± S.E. *p<0.05, compared to basal expression levels. #*p*<0.05, compared to expression levels in the adiponectin-treated group.

Because it has been reported that the ICAM-1 promoter includes binding sites for AP-1 [26], we sought to investigate whether the transcription factor is responsible for adiponectin-mediated ICAM-1 expression on OASFs observed above. Pretreatment of cells for 30 min with AP-1 inhibitors (curcumin and tanshinone IIA) antagonized adiponectin-induced ICAM-1 expression (Fig. 2A, B). We then investigated whether the presence of c-Jun and/or c-Fos is critical for adiponectin-mediated increase of ICAM-1 expression. As shown in Fig. 2C, D, the adiponectin-mediated increase of ICAM-1 expression was inhibited by knocking down c-Jun with siRNA, but not by knocking down c-fos. Next, we further examined c-Jun phosphorylation after adiponectin treatment. Stimulation of OASFs with adiponectin promoted c-Jun phosphorylation (Fig. 2E). These data indicated that AP-1 transactivation by the c-Jun homodimer is involved in adiponectin-induced ICAM-1 expression.

### The AMPK signaling pathway is involved in the adiponectin-mediated increase of ICAM-1 expression

**Figure 3:**
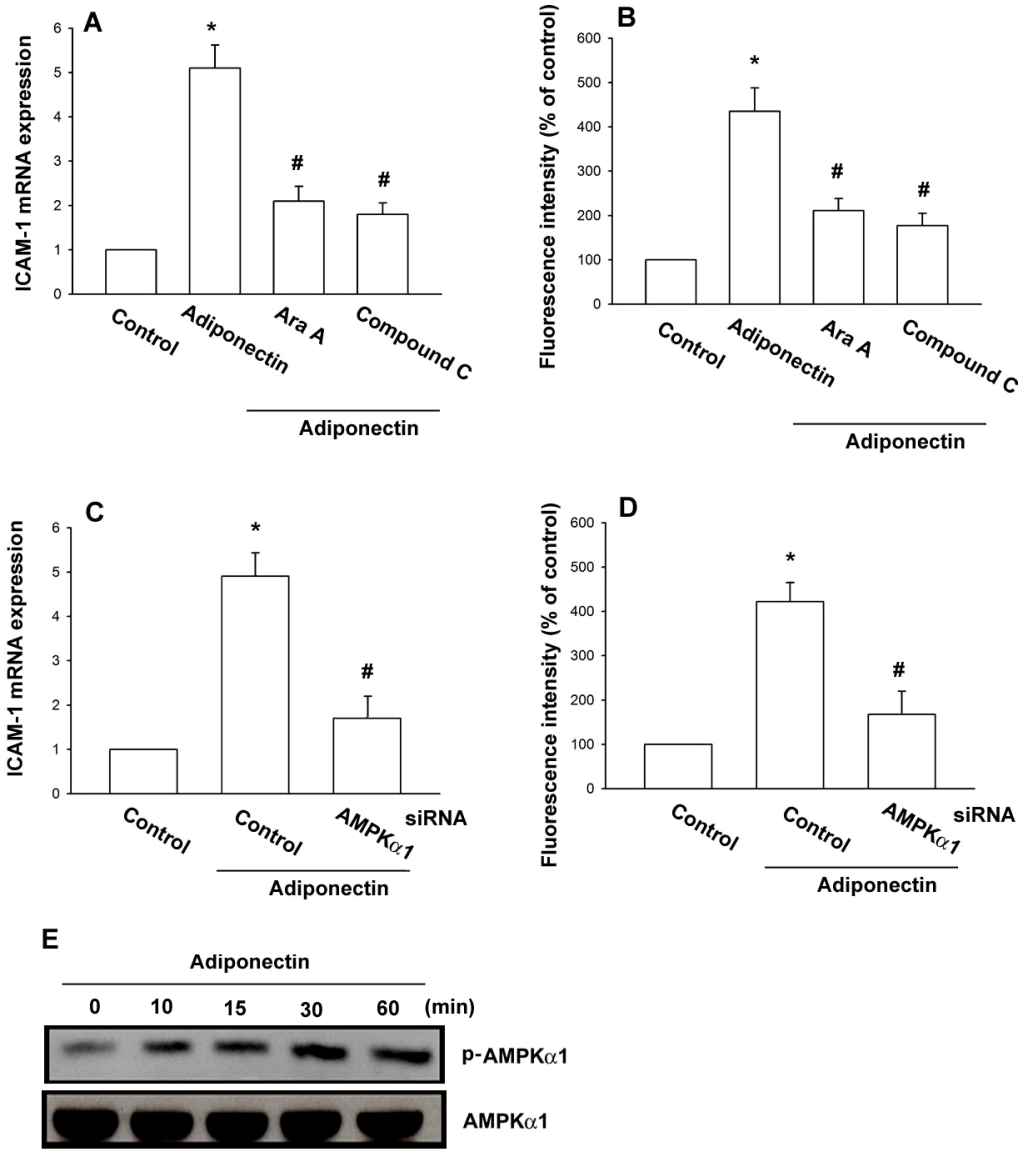
AMPK is involved in adiponectin-induced ICAM-1 expression in synovial fibroblasts. OASFs were pretreated for 30(0.5 mM) or compound C (10 μM) followed by stimulation with adiponectin (3 μg/ml) for 24 h, and ICAM-1 expression was examined by qPCR (A) and flow cytometry (B). OASFs were transfected with AMPKα1 siRNA for 24 h followed by stimulation with adiponectin (3 μg/ml) for 24 h, and ICAM-1 expression was examined by qPCR (C) and flow cytometry (D). (E) OASFs were incubated with adiponectin (3 μg/ml) for the indicated time intervals and AMPKα1 phosphorylation was determined by western blot. Results are expressed as the mean ± S.E. *p<0.05, compared to basal expression levels. #*p*<0.05, compared to expression levels in the adiponectin-treated group.

Previously studies have reported that adiponectin is able to increase fatty acid oxidation via activation of AMP-activated protein kinase (AMPK) in adipocytes [27], [28]. To determine whether AMPK is involved in adiponectin triggered ICAM-1 expression, the AMPK inhibitors Ara A and compound C were used. As seen in Fig. 3A, B, pretreatment with Ara A and compound C reduced adiponectin-induced ICAM-1 expression. Further, we knocked down the expression of AMPKα1 by its specific siRNAs in OASFs and investigated the effects of adiponectin on the ICAM-1 production. Indeed, transfection of cells with AMPKα1 siRNA diminished adiponectin-induced ICAM-1 expression (Fig. 3C, D). We then directly measured AMPK phosphorylation in response to adiponectin and found that stimulation of OASFs led to a significant increase in phosphorylation of AMPK (Fig. 3E). These data suggest that AMPK activation is involved in adiponectin-induced ICAM-1 expression in human OASFs.

### LKB1 and CaMKII signaling pathways are involved in adiponectin-induced ICAM-1 expression

**Figure 4:**
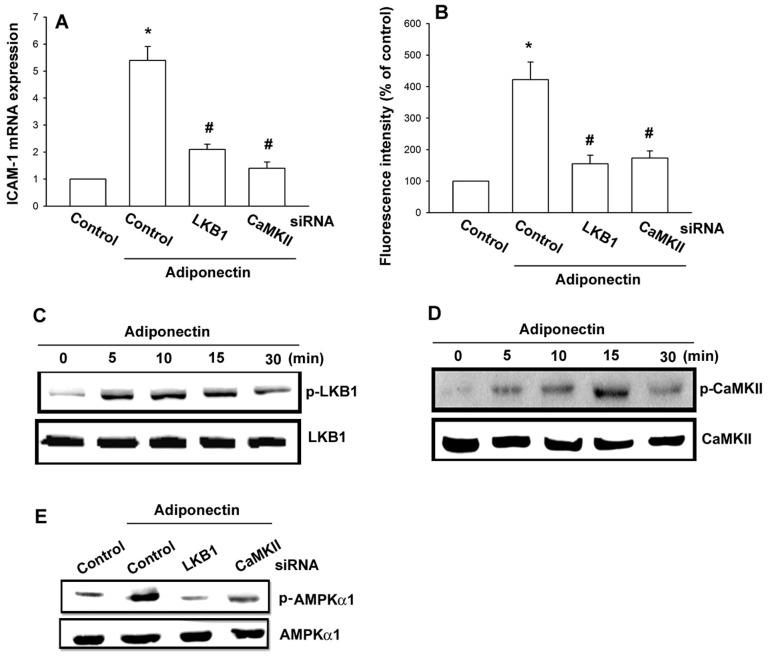
LKB1 and CaMKII are involved in adiponectin-induced ICAM-1 expression in synovial fibroblasts. OASFs were transfected with LKB1 or CaMKII siRNA for 24(3 μg/ml) for 24 h, and ICAM-1 expression was examined by qPCR (A) and flow cytometry (B). (C, D) OASFs were incubated with adiponectin (3 μg/ml) for indicated time intervals and LKB1 and CaMKII phosphorylation was determined by western blot. (E) OASFs were transfected with LKB1 siRNA or CaMKII siRNA for 24 h followed by stimulation with adiponectin (3 μg/ml) for 30 min. AMPK phosphorylation was determined by western blot. Results are expressed as the mean ± S.E. *p<0.05, compared to basal expression levels. #*p*<0.05, compared to expression levels in the adiponectin-treated group.

AMPK is regulated by upstream kinases which have been identified as LKB1 or CaMKII [29], [30]. In addition, a study indicates that adiponectin also activates AMPK upstream kinase LKB1 and CaMKII, which plays a critical role in AMPK activation [31]. To examine the role of LKB1 and CaMKII in adiponectin-mediated ICAM-1, we generated LKB1- and CaMKII- suppressed OASFs by siRNA knockdown. Both siRNA constructs significantly restricted increases in adiponectin-induced expression of ICAM-1, as determined by qPCR (Fig. 4A) and flow cytometry (Fig. 4B). Direct incubation of cells with adiponectin caused a time-dependent increase in phosphorylation of LKB1 and CaMKII (Fig. 4C, D). In addition, knockdown of LKB1 or CaMKII led to a decrease in adiponectin-induced AMPK1 phosphorylation (Fig. 4E). Taken together, these results indicate that the LKB1/CaMKII-dependent AMPK activation is involved in the regulation of ICAM-1 expression.

### LKB1/CaMKII/AMPK signaling pathway is involved in adiponectin-induced AP-1 activation

**Figure 5:**
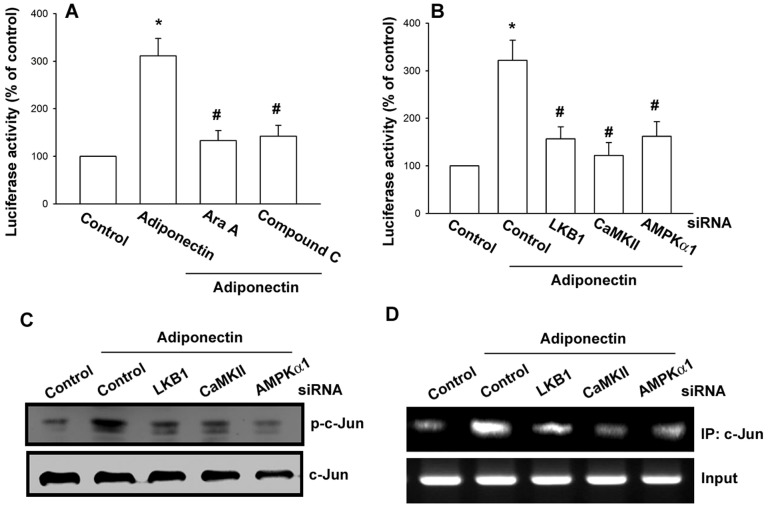
The LKB1, CaMKII, and AMPK signaling pathway is involved in adiponectin-induced AP-1 activation. OASFs transiently transfected with AP-1-luciferase plasmid for 24 h and then pretreated with Ara A and compound C (A) for 30 min or cotransfected with LKB1, CaMKII, and AMPKα1 siRNA (B) for 24 h before incubation with adiponectin for 24 h. Luciferase activity was measured, and the results were normalized to the β-galactosidase activity. OASFs were transfected with LKB1, CaMKII, or AMPKα1 siRNA for 24 h followed by stimulation with adiponectin (3 μg/ml), and c-Jun phosphorylation and c-Jun binding to the ICAM-1 promoter were examined by western blot (C) and chromatin immunoprecipitation assay (D). Results are expressed as the mean ± S.E. *p<0.05, compared to basal expression levels. #*p*<0.05, compared to expression levels in the adiponectin-treated group.

To further evaluate the LKB1/CaMKII/AMPK signaling pathway involved in adiponectin-induced AP-1 activation, OASFs were transiently transfected with AP-1 promoter-luciferase construct as an indicator of AP-1 activation. As shown in Fig. 5A, treatment of OASFs with adiponectin caused an increase in AP-1-luciferase activity, whereas pretreatment of cells with Ara A or compound C reduced adiponectin-mediated AP-1 activity. Moreover, co-transfection of cells with LKB1, CaMKII, or AMPK1 siRNA also reduced adiponectin-induced AP-1 activity (Fig. 5B)

Finally, AP-1 activation was further validated using western blot analysis and ChIP. As shown in Fig. 5C, transfection of cells with LKB1, CaMKII, and AMPK1 siRNA inhibited adiponectin-mediated c-Jun phosphorylation. ChIP analysis reveals that adiponectin significantly increased c-Jun binding to AP-1 element of ICAM-1 promoter, but this phenomenon was attenuated by transfection of cells with LKB1, CaMKII, and AMPK1 siRNA (Fig. 5D).

### Adiponectin induces monocyte adhesion through the LKB1/CaMKII/AMPK pathway

**Figure 6:**
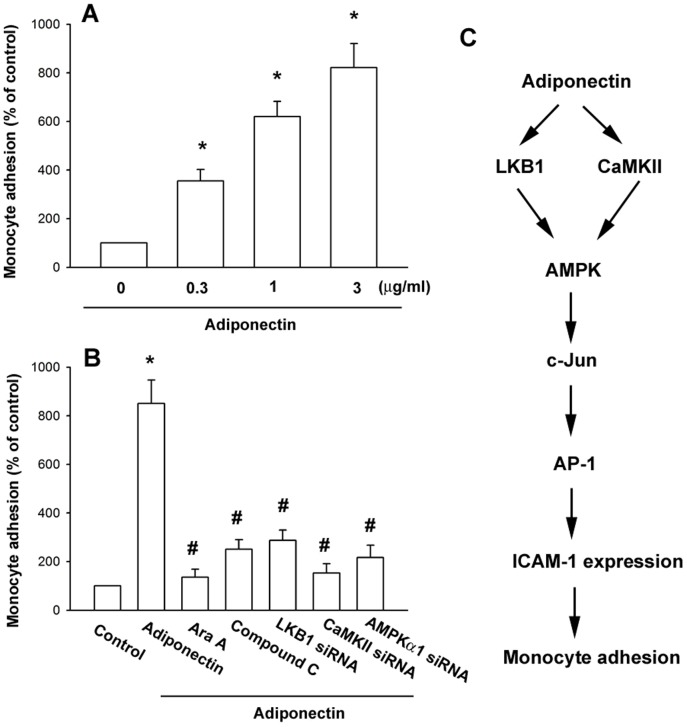
Adiponectin induces monocyte adhesion through the LKB1, CaMKII, AMPK, and AP-1 pathway. OASFs were incubated with various concentrations of adiponectin for 24(A), pretreated with Ara A and compound C for 30 min, or transfected with LKB1, CaMKII, and AMPKα1 siRNA followed by stimulation with adiponectin for 24 h (B). THP-1 cells labeled with BCECF-AM were added to OASFs for 6 h, and then the adherence of THP-1 cells was measured by fluorescence microscopy. Results are expressed as the mean ± S.E. *p<0.05, compared to basal expression levels. #*p*<0.05, compared to expression levels in the adiponectin-treated group. (C) Schematic presentation of the signaling pathways involved in adiponectin-induced ICAM-1 expression and monocyte adhesion to human synovial fibroblasts.

In order to identify whether adiponectin is involved in the interaction between OASFs and monocytes, we carried out adhesion assays using the THP-1 cell line as a monocyte model. Treatment of OASFs with adiponectin enhanced the adhesion between OASFs and THP-1 cells in a dose-dependent fashion (Fig. 6A). To further evaluate the LKB1/CaMKII/AMPK pathway is able to induce monocytes to adhere to the monolayer of OASFs, we pretreated OASFs with Ara A and compound C, and also transfected them with LKB1, CaMKII, and AMPK1 siRNA. Both the pretreatment and transfection significantly inhibited monocyte adhesion to OASFs (Fig. 6B). These results indicate that adiponectin promoted the adhesion of monocytes to OASFs via the LKB1/CaMKII/AMPK pathway.

## Discussion

OA is a chronic inflammatory disease with cytokine production that may play a key role in the recruitment and infiltration of leucocyte to the joint, resulting in degradation and loss of articular tissues. However, detailed mechanisms responsible for attracting immune cells to the site of inflamed synovium are still unclear. In this study, we characterized the effect of adiponectin on the expression of ICAM-1 in synovial fibroblasts, which may mediate the interaction of fibroblasts with immune cells. Furthermore, we also showed that potentiation of ICAM-1 by adiponectin requires the activation of the LKB1/CaMKII, AMPK, and AP-1 signaling pathway and promotes the adhesion of monocytes to OASFs (Fig. 6C).

Adiponectin is an adipocytokine originally found to be secreted exclusively by adipose tissue, but meanwhile, adiponectin was found to be expressed by bone-forming cells as well [32]. In synovial fibroblasts and articular adipocytes of rheumatoid arthritis (RA) and OA patients, high levels of adiponectin expression were also detected, which may contribute to inflammation in the fluid of the joint [11]. A previous study showed that plasma adiponectin levels were significantly higher in OA patients than in healthy controls [33]. Another study also found that increased plasma adiponectin levels were positively correlated with synovial fluid adiponectin concentrations in OA patients [9]. Adiponectin exists both as full-length and globular forms. Globular adiponectin is proteolytically cleaved from the full-length protein, which consists of the C-terminal domain of the full-length adiponectin [34]. Contrary to full-length adiponectin, globular form constitutes about 25% of adiponectin in synovial fluid from patients with arthritis [35]. Moreover, other research has shown cleavage of adiponectin by leukocyte elastase secreted from activated monocytes is able to generate the globular adiponectin [36], which may be involved in the generation of the globular fragment of adiponectin in inflamed joints. Adiponectin interacts with at least two known cellular receptors (AdipoR1 and AdipoR2). AdipoR1 is abundantly expressed in skeletal muscle, whereas AdipoR2 is predominantly expressed in the liver [37]. However, our previous studies have demonstrated that RASF and OASF cells express both AdipoR1 and AdipoR2 receptor isoforms. Furthermore, adiponectin increased IL-6 production in human synovial fibroblasts via the AdipoR1 receptor but not AdipoR2 [12]. In the present study, we found similar results. Our data indicate that adiponectin can induce ICAM-1 expression in human OASFs via AdipoR1 (Fig. S1).

Interestingly, the ability to respond to adiponectin is not exclusive to synovial fibroblasts. AdipoRs have also been identified on the surface of human chondrocytes. In chondrocytes, the binding of adiponectin to its receptor causes the increased production of IL-6, IL-8, monocyte chemoattractant protein-1 (MCP-1), prostaglandin E2, matrix metalloproteinase (MMP), and nitric oxide, which contribute to inflammation and joint destruction in OA [38]–[42]. In addition, adiponectin could elicit persistent cartilage-degrading processes by inducing expression of vascular cell adhesion molecules-1 (VCAM-1) in chondrocytes, which is responsible for infiltration of leukocyte and monocyte into inflamed joints [43]. We therefore investigated whether adiponectin can also stimulate expression of VCAM-1 on OASFs. As shown in Fig. S2A, adiponectin was also able to induce VCAM-1 expression in a dose-dependent manner. To further determine which of adhesion molecules is primarily responsible for recruitment of leukocytes on adiponectin-stimulated OASFs, we performed siRNA experiments to knock down VCAM-1 or ICAM-1 in OASFs. Our results revealed ICAM-1 kncokdown exerted more potent effect on inhibiting the adhesion of monocytes when compared with VCAM-1 kncokdown (Fig. S2B).

On the contrary, some studies have shown contradictory results. In an animal model, adiponectin has been shown to ameliorate the severity of collagen-induced arthritis [44]. Moreover, adiponectin may play a protective role against OA by inducing tissue inhibitor of metalloproteinase-2 (TIMP-2) expression and suppressing IL-1β-induced MMP-13 production in chondrocytes [45]. A clinical report has also indicated that decreased adiponectin levels in both plasma and synovial fluid is associated with severity of OA [9]. The explanation for the above discrepancies may be attributable to differences in methodologies, disease progression, populations, and inappropriate controls for normalization.

AMPK, a heterotrimeric serine/threonine kinase, consists of a catalytic α subunit, and regulatory β and γ subunits [46]. Previous studies have demonstrated that AMPK is involved in the adiponectin signaling pathway [47]–[49]. We observed that AMPK inhibitors, namely, Ara A and compound C, antagonize adiponectin-mediated ICAM-1 expression, suggesting that AMPK activation is required for adiponectin-induced ICAM-1 expression in synovial fibroblasts. In addition, it has been shown that AMPK1 is more important than AMPK2 in adiponectin-mediated gene expression in human synovial fibroblasts [12]. Thus, we attempted to investigate whether the catalytic subunit of AMPKα1 mediates adiponectin signaling in human OASFs cells. The results revealed that siRNA against AMPKα1 reduced adiponectin-mediated ICAM-1 production, implying that AMPKα1 is involved in adiponectin-induced expression of ICAM-1.

Histologically, OA synovium shows an increased number of mixed immune cells infiltrate, particularly macrophages in early OA [50]. Additionally, numerous studies have shown that activated synovial macrophages play a pivotal role in ongoing inflammation in OA through an increase in production of cytokines and destructive enzymes [51]–[53]. Another study also found that synovial fluid macrophages are capable of differentiating into mature osteoclasts to promote OA pathology [54]. The results from these reports imply that prevention of macrophage infiltration into inflamed synovium could be an attractive strategy for OA therapy.

## Conclusion

We have explored the signaling mechanisms of adiponectin in the regulation of ICAM-1 expression in human synovial fibroblasts. Our results demonstrated that adiponectin increases ICAM-1 production by activating LKB1/CaMKII and AMPK, which in turn enhances the binding of AP-1 transcription factor to the ICAM-1 promoter, leading to the transactivation of ICAM-1 expression. In addition, we also showed that the adiponectin-mediated LKB1/CaMKII, AMPK, and AP-1 pathway promotes the adhesion of monocytes to human OASFs. These findings may provide a better understanding of the mechanisms underlying OA pathogenesis and can utilize this knowledge translationally for novel treatment strategies for OA.

## Supporting Information

Figure S1 **AdipoR1, but not AdipoR2 is involved in adiponectin-induced ICAM-1 expression in synovial fibroblasts.** OASFs were transfected with AdipoR1 or AdipoR2 siRNA for 24 h followed by stimulation with adiponectin (3 μg/ml) for 24 h, and ICAM-1 expression was examined by qPCR.
(TIF)

Figure S2 **Adiponectin can also increase VCAM-1 expression to induce monocyte adhesion.** (A) OASFs were incubated with various concentrations of adiponectin for 24 h. The level of VCAM-1 mRNA was examined by qPCR. (B) OASFs were transfected with ICAM-1 or VCAM-1 siRNA for 24 h followed by stimulation with adiponectin (3 μg/ml) for 24 h. THP-1 cells labeled with BCECF-AM were added to OASFs for 6 h, and then the adherence of THP-1 cells was measured by fluorescence microscopy. Results are expressed as the mean ± S.E. *p<0.05, compared to basal expression levels. #*p*<0.05, compared to expression levels in the adiponectin-treated group.
(TIF)
