# Supplementary figures and images for: Adiponectin Enhances Intercellular Adhesion Molecule-1 Expression and Promotes Monocyte Adhesion in Human Synovial Fibroblasts
Source: PLoS One. 2014 Mar 25;9(3):e92741. doi: 10.1371/journal.pone.0092741 (PMC3965461; doi:10.1371/journal.pone.0092741)

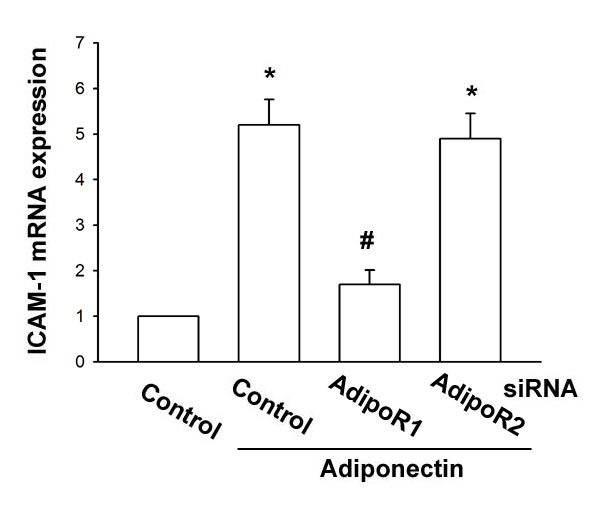

Supplement: Figure S1 — AdipoR1, but not AdipoR2 is involved in adiponectin-induced ICAM-1 expression in synovial fibroblasts. OASFs were transfected with AdipoR1 or AdipoR2 siRNA for 24 h followed by stimulation with adiponectin (3 μg/ml) for 24 h, and ICAM-1 expression was examined by qPCR. (TIF) [file pone.0092741.s001.tif]

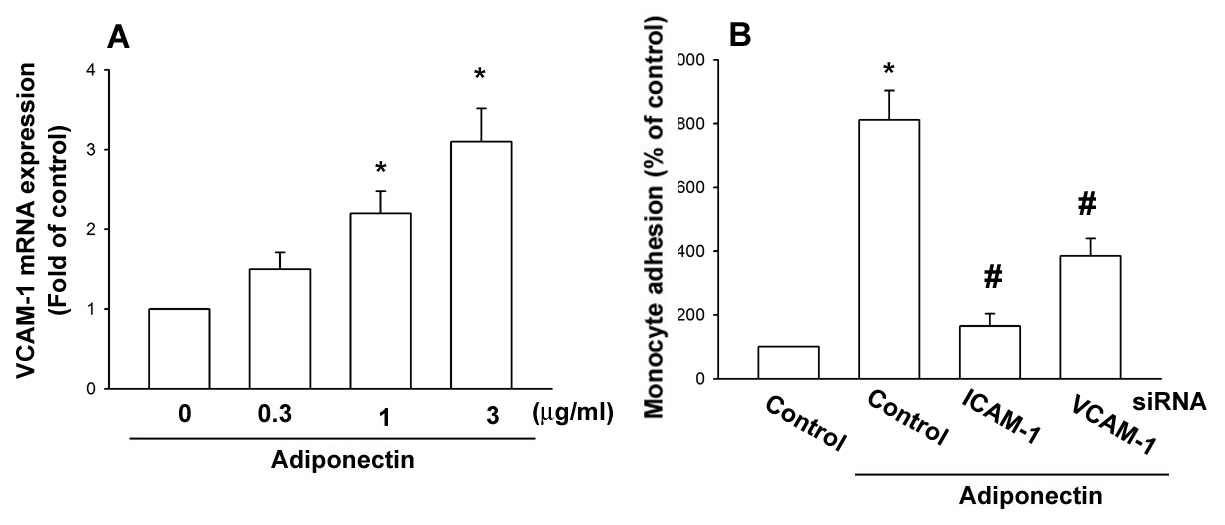

Supplement: Figure S2 — Adiponectin can also increase VCAM-1 expression to induce monocyte adhesion. (A) OASFs were incubated with various concentrations of adiponectin for 24 h. The level of VCAM-1 mRNA was examined by qPCR. (B) OASFs were transfected with ICAM-1 or VCAM-1 siRNA for 24 h followed by stimulation with adiponectin (3 μg/ml) for 24 h. THP-1 cells labeled with BCECF-AM were added to OASFs for 6 h, and then the adherence of THP-1 cells was measured by fluorescence microscopy. Results are expressed as the mean ± S.E. *p<0.05, compared to basal expression levels. #p<0.05, compared to expression levels in the adiponectin-treated group. (TIF) [file pone.0092741.s002.tif]
